# Liraglutide Attenuates Hepatic Ischemia–Reperfusion Injury by Modulating Macrophage Polarization

**DOI:** 10.3389/fimmu.2022.869050

**Published:** 2022-04-05

**Authors:** Shang-Lin Li, Zhi-Min Wang, Cong Xu, Fu-Heng Che, Xiao-Fan Hu, Rui Cao, Ya-Nan Xie, Yang Qiu, Hui-Bo Shi, Bin Liu, Chen Dai, Jun Yang

**Affiliations:** ^1^Institute of Organ Transplantation, Tongji Hospital, Tongji Medical College, Huazhong University of Science and Technology, Wuhan, China; ^2^Key Laboratory of Organ Transplantation, Ministry of Education, NHC Key Laboratory of Organ Transplantation, Key Laboratory of Organ Transplantation, Chinese Academy of Medical Sciences, Wuhan, China; ^3^Department of Pediatrics, Tongji Hospital, Tongji Medical College, Huazhong University of Science and Technology, Wuhan, China

**Keywords:** acute liver injury, ischemia-reperfusion, liraglutide, macrophage polarization, glucagon-like peptide-1 receptor

## Abstract

Ischemia-reperfusion injury (IRI) is a common complication associated with liver surgery, and macrophages play an important role in hepatic IRI. Liraglutide, a glucagon-like peptide-1 (GLP-1) analog primarily used to treat type 2 diabetes and obesity, regulates intracellular calcium homeostasis and protects the cardiomyocytes from injury; however, its role in hepatic IRI is not yet fully understood. This study aimed to investigate whether liraglutide can protect the liver from IRI and determine the possible underlying mechanisms. Our results showed that liraglutide pretreatment significantly alleviated the liver damage caused by ischemia-reperfusion (I/R), as evidenced by H&E staining, serum aspartate aminotransferase (AST) and alanine aminotransferase (ALT) levels, and TUNEL staining. Furthermore, the levels of inflammatory cytokines elicited by I/R were distinctly suppressed by liraglutide pretreatment, accompanied by significant reduction in TNF-α, IL-1β, and IL-6 levels. Furthermore, pretreatment with liraglutide markedly inhibited macrophage type I (M1) polarization during hepatic IRI, as revealed by the significant reduction in CD68^+^ levels in Kupffer cells (KCs) detected *via* flow cytometry. However, the protective effects of liraglutide on hepatic IRI were partly diminished in GLP-1 receptor-knockout (GLP-1R^-/-^) mice. Furthermore, in an *in vitro* study, we assessed the role of liraglutide in macrophage polarization by examining the expression profiles of M1 in bone marrow-derived macrophages (BMDMs) from GLP-1R^-/-^ and C57BL/6J mice. Consistent with the results of the *in vivo* study, liraglutide treatment attenuated the LPS-induced M1 polarization and reduced the expression of M1 markers. However, the inhibitory effect of liraglutide on LPS-induced M1 polarization was largely abolished in BMDMs from GLP-1R^-/-^ mice. Collectively, our study indicates that liraglutide can ameliorate hepatic IRI by inhibiting macrophage polarization towards an inflammatory phenotype *via* GLP-1R. Its protective effect against liver IRI suggests that liraglutide may serve as a potential drug for the clinical treatment of liver IRI.

## Introduction

Hepatic IRI is a common clinical, pathophysiological phenomenon that often occurs during trauma, shock, liver surgery, and liver transplantation. It is the main factor affecting the complications associated with liver surgery and the survival and prognosis of patients (1, 2). In addition, liver dysfunction caused by hepatic ischemia-reperfusion injury can further affect distal organs and cause systemic damage (3). Although liver IRI involves a variety of molecular mechanisms and results from the joint action of various cells, its underlying mechanism has not yet been fully elucidated.

Recent studies have shown that macrophages play an important role in hepatic IRI (4, 5). Hepatic IRI can be divided into early and late stages. The early stage of IRI involves the rapid activation of KC cells after reperfusion, and the late stage is characterized by the recruitment of neutrophils to the liver (6). As the largest immune organ in the human body, the liver contains various immune cells such as resident macrophages (KCs), dendritic cells (DCs), natural killer cells (NKs), and natural killer T cells (NKTs) (7). In these cells, the macrophage-mediated inflammatory response is considered an important factor in hepatic IRI. Liver macrophages are divided into M1-and M2-type macrophages according to their phenotype and function. M1 macrophages promote the development of inflammation, whereas M2 macrophages inhibit inflammation. The proportion of M1/M2 macrophages in the liver affects the pathological outcome of hepatic IRI (8).

Liraglutide, a glucagon-like peptide-1 (GLP-1) analog that binds to and activates GLP-1R, is primarily used to treat type 2 diabetes and obesity (9, 10). Recent studies have shown that GLP-1R is distributed in the pancreas and organs such as the heart, lung, liver, and kidney (11, 12). In addition to regulating blood sugar levels, liraglutide has other biological activities as well. For example, it can regulate intracellular calcium homeostasis to protect cardiomyocytes from injury (13). In addition, liraglutide has been reported to play a role in liver protection by reducing inflammation in a liver/nonalcoholic steatohepatitis (NAFLD/NASH) model (14).

This study aimed to examine the protective effect of liraglutide on hepatic IRI and its effects on macrophages and explore the possible underlying mechanism using a partial hepatic IRI mouse model. The outcomes of this study could provide a new direction for the clinical treatment of HIRI.

## Materials and Methods

### Mice

C57BL/6J (B6) mice were purchased from Beijing HFK Bioscience Co. Ltd. (Beijing, China). GLP-1R^-/-^ mice were purchased from Beijing Biocytogen Co. Ltd. (Beijing, China). All mice were 6–8 weeks of age and housed under specific pathogen-free conditions at the Tongji Medical School Facilities for Animal Care and Housing. All animal studies were performed in accordance with the guidelines of the Chinese Council on Animal Care and approved by the Tongji Medical College Committees on Animal Experimentation.

### Reagents and Experimental Design

Liraglutide was purchased from Novo Nordisk (Copenhagen, Denmark) and diluted in 0.9% normal saline before administration. Each 200 μg/kg dose of liraglutide was subcutaneously administered every 12 h for three consecutive days to the mice. The dose of liraglutide was based on our previous study (15).

In the GdCl_3_ treatment group, mice were injected intravenously with GdCl_3_ at 10 mg/kg (Sigma-Aldrich, USA) 24 h before the onset of liver ischemia. For NK and NKT cell depletion, anti-NK1.1 MAb (PK136, BioLegend, USA) was intraperitoneally injected (200 ug per mouse) on day -2, -1, 0 before the procedure. For depletion of NK cells alone, 50 µg of anti-asialoGM1 Ab (Sigma-Aldrich, USA) was intraperitoneally injected twice on day -3 and -1 before liver IRI. In addition, anti-Gr-1 (BioLegend, USA) was intraperitoneally injected (10 mg/kg) 24 h prior to hepatic IRI.

### Liver IRI Model

Male C57BL/6J wild-type and GLP-1R^-/-^ mice (6–8 weeks of age) were used in these experiments. The partial liver I/R animal model was established as described in our previous publication, with some modifications (16). Briefly, mice were anesthetized with sodium pentobarbital (80 mg/kg, intraperitoneally) (Sigma, MO, USA), and after midline laparotomy, the arterial and portal venous blood supplies to the left and middle liver lobes were interrupted with an atraumatic clip (using an operating microscope) for 60 min of partial hepatic warm ischemia, and the clamp was then removed. Body temperature was maintained at 32°C using a warming pad. The sham animals underwent the same procedures but were not subjected to hepatic ischemia. After a certain time (2, 6, or 24 h) of reperfusion, the mice were sacrificed for tissue analysis.

### Isolation of Kupffer Cells (KCs)

KCs were isolated as previously described by Li et al. (17). Briefly, livers were perfused with 10 mL of calcium-free Hank balanced salt solution (HyClone Laboratories, USA) *via* the portal vein, followed by 0.27% type IV collagenase (Sigma-Aldrich, USA) in a water bath at 37°C for 20 min. The perfused liver was dissected, and a 70 mm cell filter was used to produce a single-cell suspension. Next, KCs were obtained by discontinuous density gradient centrifugation. After 2 h of incubation at 37°C and 5% CO_2_, the isolated KCs were purified by removing the non-adherent cells.

### Extraction and Culture of Bone Marrow-Derived Macrophages (BMDMs)

BMDMs were obtained from fresh BM cells of C57BL/6J mice or GLP-1R^-/-^ mice. These cells were then cultured in a DMEM medium (Gibco, China) containing 10% heat-inactivated fetal bovine serum (FBS) and granulocyte-macrophage colony-stimulating factor (GM-CSF, PeproTech, USA, 10 ng/ml). The medium was replaced every two days. Five days later, the cells were pretreated with liraglutide (50 μM) for 24 h and then treated with LPS (100ng/ml) for 24 h.

### Serum Transaminase Level Measurement

Blood samples were collected at a particular time, and serum alanine aminotransferase (ALT) and aspartate aminotransferase (AST) levels were measured using an automated biochemical analyzer BS-200 (Mindray, Shenzhen, China).

### Histopathology Assay

Harvested liver tissues were fixed in 4% formalin and embedded in paraffin blocks. Four-micrometer sections were stained with hematoxylin and eosin (H&E), and liver damage caused by I/R was scored by a pathologist blindly using Suzuki criteria (18) on a scale from 0–4.

### Immunofluorescence and Immunohistochemical Assays

For immunofluorescence analysis, paraffin-embedded liver sections were incubated with primary antibodies against F4/80 (1:3000, Servicebio, China) and iNOS (1:200, Abcam, USA), followed by incubation with a secondary antibody (Servicebio, Wuhan, China). Apoptotic cells were evaluated using the terminal deoxynucleotidyl transferase (TdT) dUTP nick-end labeling (TUNEL) assay kit (Roche Applied Science) according to a standard protocol. Liver myeloperoxidase (MPO) activity was assessed using immunohistochemistry. The sections were incubated with primary antibodies against MPO (Servicebio, Wuhan, China), followed by the application of the appropriate secondary antibody (Servicebio, Wuhan, China). Five microscopic fields were examined for each section and used for calculations.

### Flow Cytometric Analysis

BMDMs and KCs were stained with monoclonal antibodies against F4/80 APC, CD11b BV421, CD68 FITC and CD86 BV605 (eBioscience, San Diego, CA, USA) at 4°C for 30 min, washed with phosphate-buffered saline and fixed with 1% formalin. Flow cytometry analysis was performed using a FACSCaliber system (BD Biosciences, USA) and analyzed using the FlowJo software.

### Western Blotting

Proteins were extracted from the I/R liver and cultured BMDMs for western blotting. The primary antibody used was anti-STAT1 (1:1000; CST, USA), anti-Bax (1:1000; ABclonal, Wuhan, China), anti-Bcl-2 (1:1000; ABclonal, China), and anti-β-actin (1:10000; ProteinTech, USA). Membranes were then incubated with horseradish peroxidase-conjugated goat anti-rabbit polyclonal secondary antibodies (1:1000; Servicebio, Wuhan, China). The protein bands were visualized using GeneGnome XRQ (SYNGENE, UK), and data were obtained from three independent experiments.

### Quantitative Reverse Transcription-PCR (qRT-PCR)

Total RNA was extracted from mouse livers or BMDMs using TRIzol reagent (Ambion) and reverse-transcribed into cDNA templates using a PrimeScriptTM RT reagent kit (Takara, Japan). RT-qPCR was performed using SYBR^®^ Premix Ex TaqTM II (Tli RNaseH Plus) (Takara, Japan) with the following primers: iNOS (forward: 5′-ATTCACAGCTCATCCGGTACG-3′; reverse: 5′-GGATCTTGACCATCAGCTTGC-3′), TNF-α (forward: 5′-TATGGCTCAGGGTCCAACTC-3′, reverse: 5′-GGAAAGCCCATTTGAGTCCT-3′), IL-6 (forward: 5′-ACCAGAGGAAATTTTCAATAGGC-3′, reverse: 5′-TGATGCACTTGCAGAAAACA-3′), IL-1β (forward: 5′-GGTCAAAGGTTTGGAAGCAG-3′, reverse: 5′-TGTGAAATGCCACCTTTTGA-3′), and GLP-1R (forward: 5′-ACAGTGGGGTACGCACTTTC-3′, reverse: 5′-CGGAGGATGAAGGATGCAAAC-3′), and GAPDH (forward: 5′-AGGTCGGTGTGAACGGATTTG-3′, reverse: 5′-TGTAGACCATGTAGTTGAGGTCA-3′). Samples were analyzed in triplicate using StepOne Software v2.3 (Thermo Fisher Scientific, USA). For quantitative analysis, all samples were analyzed using the ΔΔCT method.

### Statistical Analysis

All data are expressed as mean ± standard deviation (SD). Student’s unpaired t-test was used to compare the differences in the means between two groups, and one-way analysis of variance (ANOVA) followed by Bonferroni’s *post hoc* test was used to perform multiple statistical comparisons. Statistical significance was set at P < 0.05. All statistical analyses were performed using GraphPad Prism 8.

## Results

### Liraglutide Pretreatment Ameliorates I/R-Induced Liver Damage in Mice

**Figure 1:**
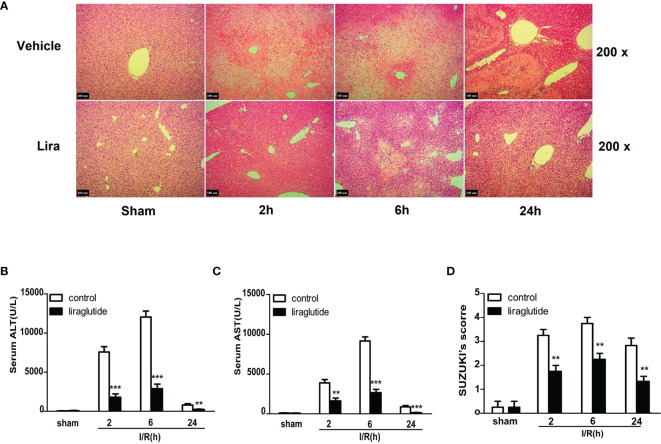
Liraglutide pretreatment ameliorates I/R-induced liver damage. Mice were subjected to 60 min of hepatic ischemia, followed by 2, 6, and 24 h of reperfusion. **(A)** H&E staining was performed to assess live injury in mice (original magnification 200×). **(B, C)** Serum ALT and AST levels of the control and liraglutide-treated mice were detected after hepatic IRI (n = 4–6 per group). **(D)** The degree of liver injury was assessed by Suzuki’s injury score (n = 3 per group). These results were obtained from at least three independent experiments. Values are presented as mean ± SD. **P < 0.01, ^***^P < 0.001 vs. the control group.

A mouse partial liver IRI model with different reperfusion time intervals was established to investigate the protective effects of liraglutide on hepatic IRI. Serum and liver specimens were harvested 2, 6, and 24 h after reperfusion, and liver injury was assessed by hematoxylin and eosin (H&E) staining (**Figure 1A**), serum ALT (**Figure 1B**), and AST (**Figure 1C**) levels. As shown in **Figure 1B**, ALT and AST levels gradually increased 2 and 6 h after reperfusion in the I/R group. However, liver function gradually recovered 24 h after reperfusion, and ALT and AST values were significantly attenuated in the liraglutide pretreatment group at different reperfusion times (**Figures 1B, C**, P < 0.01 or P < 0.001). Consistent with these results, H&E staining (**Figure 1A**) showed that the hepatic sinusoids were severely congested and swollen with extensive necrosis and a large number of infiltrating lymphocytes after reperfusion. Liraglutide pre-treatment distinctly improved these pathological changes at different reperfusion time intervals, verified by the Suzuki score (**Figure 1D**, P < 0.01). Overall, our results demonstrated that liraglutide pre-treatment protected against hepatic IRI.

### Liraglutide Pretreatment Attenuates the Apoptosis of Liver Cells Caused by I/R

**Figure 2:**
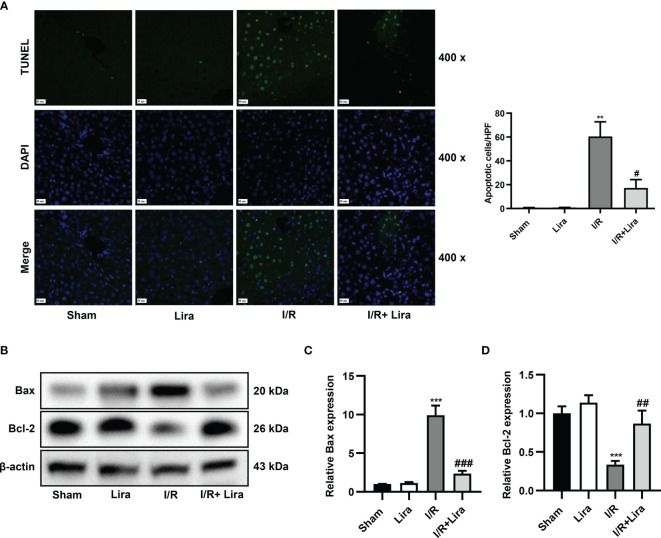
Liraglutide pretreatment attenuates the apoptosis of liver cells caused by I/R. Mice were subjected to 60 min of hepatic ischemia, followed by 6 h of reperfusion. **(A)** TUNEL staining was performed, and TUNEL-positive cells were calculated (original magnification 400×). **(B)** The expression of Bax and Bcl-2 was determined *via* western blotting. Densitometric analysis of the Bax/β-actin **(C)**, Bcl-2/β-actin **(D)** ratios is shown. All the results were obtained from at least three independent experiments. Data are presented as mean ± SD, n = 3 per group. ^**^P < 0.01, ^***^P < 0.001 vs. the Sham group; ^#^P < 0.05, ^##^P < 0.01, and ^###^P < 0.001 vs. the I/R group.

Hepatocyte apoptosis in I/R-induced liver injury was assessed using TUNEL staining and western blotting. As demonstrated in **Figure 2A**, the number of TUNEL-positive cells significantly increased 6 h after reperfusion, whereas apoptosis was distinctly reduced in the liraglutide treatment group (P < 0.05). The balance between the pro-apoptotic protein Bax and anti-apoptotic protein Bcl-2 determines whether cells survive or undergo apoptosis. Hepatic IRI caused a significant increase in Bax expression and a marked reduction in Bcl-2 at the protein level, and liraglutide pretreatment partially reversed these changes (**Figures 2B–D**, P < 0.01 or P < 0.001). These results indicate that liraglutide pretreatment alleviated I/R-induced apoptosis in the liver.

### The Protective Effect of Liraglutide Against Hepatic IRI May Be Realized Through Its Action on Macrophages

**Figure 3:**
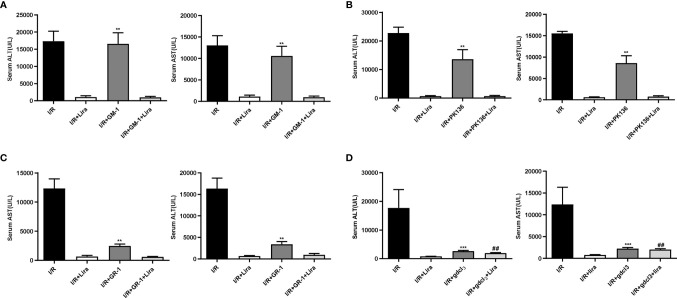
The protective effect of liraglutide on hepatic IRI may be realized through its action on macrophages. NKs, NKTs, neutrophils, and macrophages were eliminated before liver IRI. Serum ALT and AST levels of each group after NK **(A)**, NKT **(B)**, neutrophil **(C),** and macrophage **(D)** depletion. Similar results were obtained from at least three independent experiments. Data are shown as mean ± SD, n = 3–6 per group. ^**^P < 0.01, ^***^P < 0.001 vs. the I/R+Lira group; ^##^P < 0.01 vs. the I/R+Lira group. NKs, natural killer cells; NKTs, natural killer T cells.

The liver contains many immune cells such as resident macrophages (KCs), neutrophils, natural killer cells (NKs), and natural killer T cells (NKTs). To investigate the effect of liraglutide on liver IRI, we eliminated these cells from mice. Surprisingly, liraglutide exerted hepatoprotective effects after the depletion of NKs (**Figure 3A**), NKTs (**Figure 3B**), and neutrophils (**Figure 3C**). However, after removing macrophages (**Figure 3D**, P < 0.01), the protective effects of liraglutide on liver IRI were largely abolished. These results demonstrated that macrophages might mediate the protective effect of liraglutide against liver IRI.

### Liraglutide Pretreatment Inhibits I/R-Elicited M1 Polarization of Kupffer Cells in the Liver

**Figure 4:**
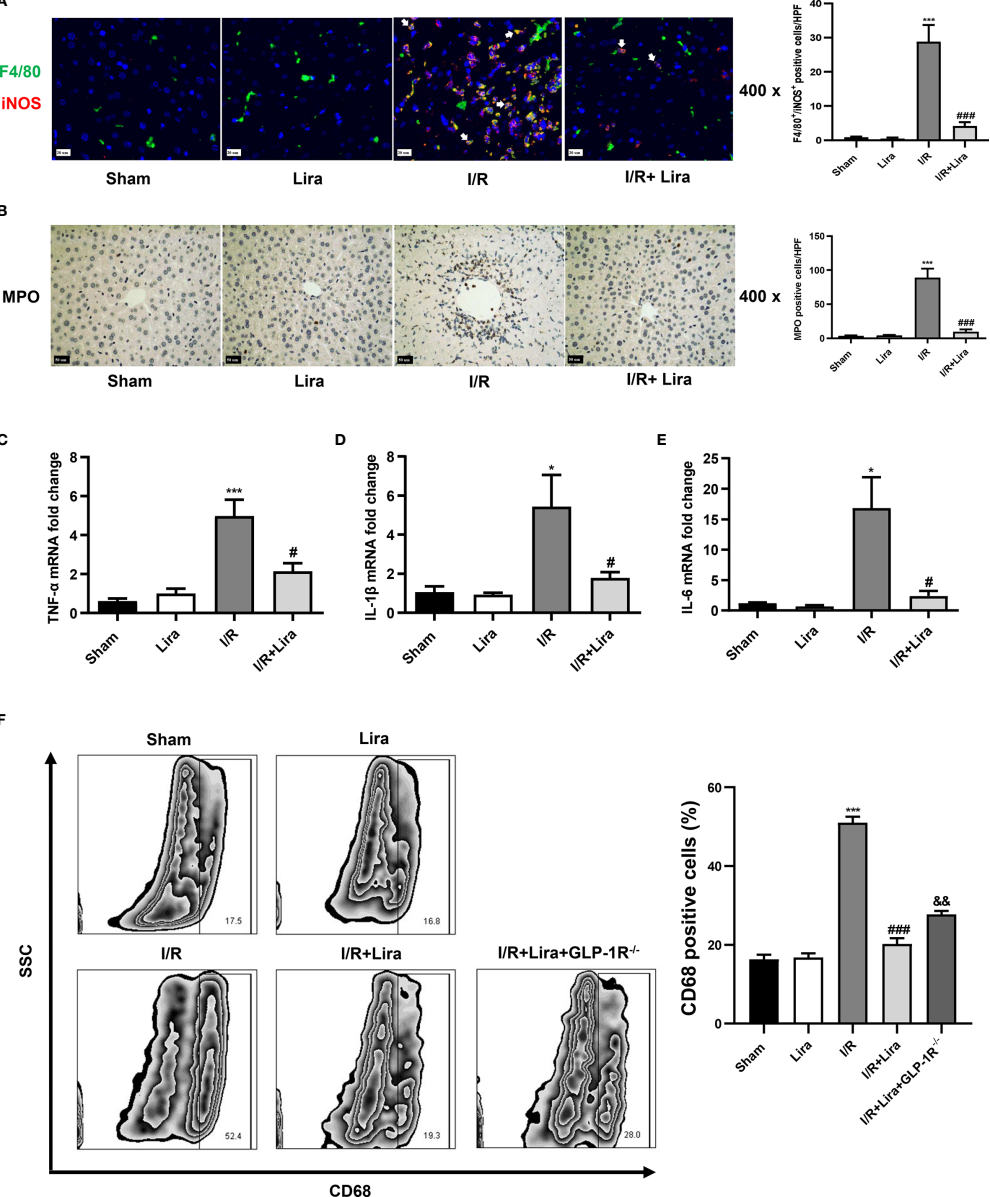
Liraglutide pretreatment inhibits I/R-elicited M1 polarization of KCs in the liver. Mice underwent 60 min of liver ischemia and 6 h of reperfusion; liver tissues were harvested for immunofluorescence staining **(A)** and MPO staining **(B)** (original magnification 400×), and the number of F4/80^+^/iNOS^+^ positive cells and MPO-positive cells was calculated. The expression of pro-inflammatory cytokines, including TNF-α **(C)**, IL-1β **(D)**, and IL-6 **(E),** in the liver was detected *via* qRT-PCR. **(F)** The number of CD68^+^-positive cells of KCs under F4/80^+^ and CD11b^+^ subsets was calculated *via* flow cytometry. The results represent the means from at least 3 independent experiments. Values are shown as mean ± SD, n = 3 per group. ^*^P < 0.05, ^***^P < 0.001 vs. the Sham group; ^#^P < 0.05, ^###^P < 0.001 vs. the I/R group. ^&&^P < 0.01 vs. the I/R+Lira group. KCs, resident macrophages; MPO, myeloperoxidase.

Previous studies have shown that macrophage polarization plays an important role in hepatic IRI (19). Activated M1 macrophages can aggravate the inflammatory response by secreting various pro-inflammatory cytokines such as TNF-α, IL-1β, and IL-6. Therefore, we further investigated the effects of liraglutide on macrophages during liver IRI. First, macrophage phenotyping was performed by dual immunofluorescence using the macrophage markers F4/80 and M1-induced nitric oxide synthase (iNOS) markers. As presented in **Figure 4A**, more macrophages in the I/R group showed M1-type macrophages, as evidenced by an increase in F4/80^+^/iNOS^+^ positive cells, whereas liraglutide pretreatment markedly suppressed M1 polarization induced by I/R (P < 0.001). Next, we evaluated neutrophil infiltration and M1 markers using MPO staining and qRT-PCR, respectively. In the I/R group, MPO-positive cells, TNF-α, IL-1β and IL-6 levels were increased in the liver. In contrast, liraglutide treatment significantly reduced these I/R-induced elevations (**Figures 4B–E**, P < 0.05 or P < 0.001).

KCs were isolated from the liver to verify these results, and CD68^+^ positive cells in the F4/80^+^ and CD11b^+^ subsets were calculated by flow cytometry. According to immunohistochemical staining results, liraglutide significantly decreased M1 polarization during liver IRI. As shown in **Figure 4F**, the percentage of CD68^+^ cells was significantly lower in the I/R+Lira group than in the I/R group (52.4.0% vs. 19.3%, P < 0.001). However, in GLP-1R^-/-^ mice, the decrease in CD68^+^ cells was suppressed (28.0% vs. 19.3%, P < 0.01). These results confirmed our findings that liraglutide pretreatment reduced M1 macrophages during hepatic IRI, and GLP-1R probably mediated the protective effects of liraglutide on liver IRI. Overall, these results showed that liraglutide pretreatment reduced the inflammatory response by inhibiting I/R-elicited M1 polarization in the liver.

### Liraglutide Pretreatment Reduces LPS-Induced M1 Polarization *In Vitro*

**Figure 5:**
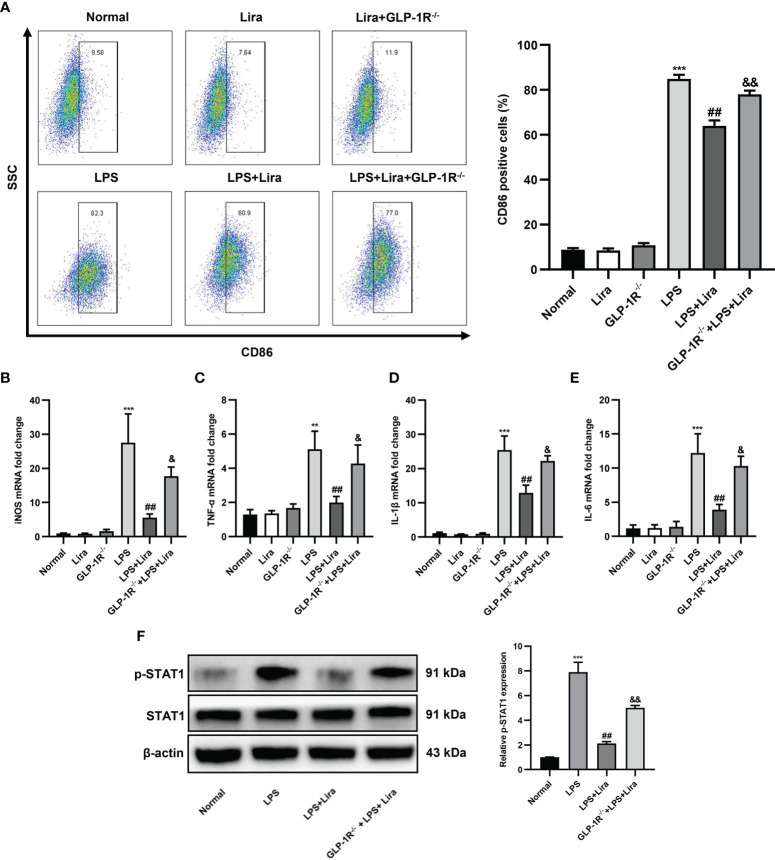
Liraglutide pretreatment reduces LPS-induced M1 polarization *in vitro*. BMDMs from GLP-1R^-/-^ and C57BL/6J mice were isolated and cultured for *in vitro* study. The cells were pretreated with liraglutide (50 µm) for 24 h, stimulated with LPS (100 nm) for 24 h, and the expression profiles of M1 in BMDMs in each group were analyzed *via* flow cytometry, RT-PCR, and western blotting. **(A)** The number of CD86^+^-positive cells of BMDMs under F4/80^+^ and CD11b^+^ subsets was calculated *via* flow cytometry. The mRNA levels of iNOS **(B)**, TNF-α **(C)**, IL-1β **(D)**, and IL-6 **(E)** were measured *via* qRT-PCR. **(F)** The phosphorylation of STAT1 was analyzed *via* western blotting. Similar results were obtained from at least three independent experiments. Data are expressed as mean ± SD, n = 3 per group. ^**^P < 0.01, ^***^P < 0.001 vs. the Normal group; ^##^P < 0.01 vs. the LPS group. ^&^P < 0.05, ^&&^P < 0.01 vs. the LPS+Lira group. BMDMs, bone marrow derived macrophages.

Next, we investigated the role of liraglutide in regulating macrophage M1 polarization. BMDMs from GLP-1R^-/-^ and C57BL/6J mice were isolated and cultured for *in vitro* studies. After stimulation with LPS for 24 h, the expression profiles of M1 in BMDMs were analyzed using flow cytometry and qRT-PCR. As shown in **Figure 5A**, the ratio of CD86^+^ positive cells was significantly increased in the LPS-treated group, while M1 phenotypic transformation was reduced by liraglutide pretreatment (**Figure 5A**, second row: 82.3% vs. 60.9%, P < 0.01), and this inhibitory effect was partly diminished in BMDMs from GLP-1R^-/-^ mice (**Figure 5A**, second row, 77.0% vs. 60.9%, P < 0.01). Pursuant to the above results, the expression profiles of LPS-induced M1, such as iNOS (**Figure 5B**, P < 0.01), TNF-α (**Figure 5C**, P < 0.01), IL-1β (**Figure 5D**, P < 0.01), and IL-6 (**Figure 5E**, P < 0.01), were obviously decreased by liraglutide pretreatment. However, the inhibitory effect of liraglutide on LPS-induced M1 phenotypic transformation was reversed in BMDMs from GLP-1R^-/-^ mice (**Figures 5B–E**, P < 0.05).

Macrophage polarization is a complex process involving stimulus recognition and the activation of transcription factors (20). Recent studies have suggested that the STAT1 signaling pathway is involved in M1 macrophage polarization (21). To investigate whether liraglutide affects these cascades, we examined STAT1 phosphorylation by western blotting. Compared to unstimulated cells, the phosphorylation of STAT1 was increased in response to LPS stimulation. However, liraglutide pretreatment significantly inhibited LPS-induced phosphorylation of STAT1, and this inhibitory effect of liraglutide on LPS-induced phosphorylation of STAT1 was partly abolished in BMDMs from GLP-1R^-/-^ mice (**Figure 5F**, P < 0.01). In summary, our results derived from GLP-1R-KO and C57BL/6J mice clearly indicated that the regulation of macrophage polarization by liraglutide is dependent on the GLP-1R receptor.

### The Protective Effects of Liraglutide Against Hepatic IRI Are Partly Diminished in *GLP-1R*-Knockout Mice

**Figure 6:**
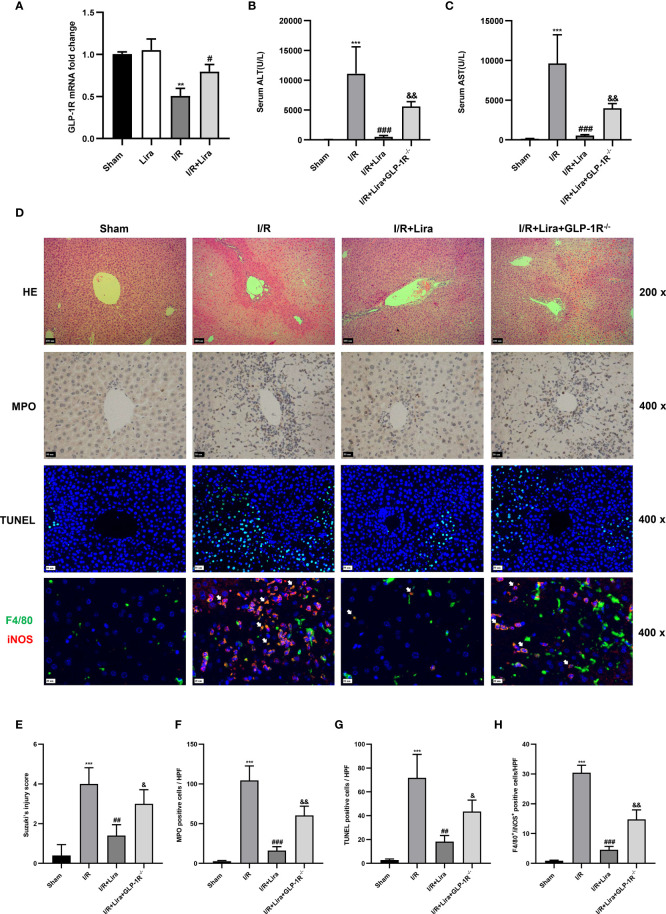
The protective effects of liraglutide on hepatic IRI are partly diminished in *GLP-1R*- knockout mice. A mouse liver partial IRI model was established using C57BL/6J mice and GLP-1R^-/-^ mice. **(A)**qRT-PCT was performed to detect GLP-1R expression in KCs from sham-operated, Lira-treated, I/R, and I/R+ Lira-treated groups (n = 3 per group). Serum levels of ALT **(B)** and AST **(C)** in each group (n = 4–6 per group). **(D)** Liver injury, neutrophil infiltration, hepatocyte apoptosis, and macrophage phenotyping were assessed *via* H&E staining, MPO, TUNEL assays, and immunofluorescence staining, respectively. Quantitation of liver injury **(E)**, neutrophil infiltration **(F)**, hepatocyte apoptosis **(G)**, and macrophage phenotyping **(H)** in each group (n = 3 per group). These results were obtained from at least three independent experiments. All values are expressed as mean ± SD. ^**^P < 0.01, ^***^P < 0.001 vs. the Sham group; ^#^P < 0.05, ^##^P < 0.01, and ^###^P < 0.001 vs. the I/R group. ^&^P < 0.05, ^&&^P < 0.01 vs. the I/R+Lira group.

The biological effects of liraglutide, a GLP-1R agonist, are mainly mediated by GLP-1R (22). Therefore, we examined the expression of GLP-1R in KCs exposed or unexposed to I/R using qRT-PCR. As expected, there is a certain expression of GLP-1R in the sham and liraglutide treatment groups (**Figure 6A**), whereas a significant reduction in GLP-1R expression was detected in the I/R group. However, liraglutide pretreatment markedly prevented the decrease in KC expression of GLP-1R at 6 h after reperfusion (**Figure 6A**, P < 0.05).

To determine whether the protective effects of liraglutide on hepatic IRI depended on GLP-1R, a mouse liver partial IRI model was used in C57BL/6J and GLP-1R^-/-^ mice. Compared with the I/R group, liraglutide pretreatment significantly reduced the serum ALT and AST levels (**Figures 6B, C**, P < 0.001). However, when subjected to the same liraglutide treatment and hepatic I/R procedure, GLP-1R^-/-^ mice showed moderately elevated serum ALT and AST levels (**Figures 6B, C**, P < 0.01). In addition, the inhibitory effects of liraglutide on liver injury, neutrophil infiltration, hepatocyte apoptosis, and M1 polarization were partly diminished in GLP-1R^-/-^ mice (**Figures 6D–H**).

Overall, these results indicate that the protective effects of liraglutide on hepatic IRI were partly dependent on GLP-1R.

## Discussion

Hepatic IRI widely exists in clinical environments, such as liver resection, liver transplantation, and trauma, and is a key factor affecting postoperative liver function, leading to liver dysfunction and even failure (23). Therefore, the prevention and treatment of hepatic IRI are common concerns for many clinicians. With an in-depth study of hepatic IRI, the role of macrophage polarization in hepatic IRI has gradually been recognized. In the early stage of liver IRI, liver macrophages mainly show the M1-dominated pro-inflammatory response, while in the late stage of liver IRI, liver macrophages primarily exhibit M2-dominated promotion of tissue repair (24). Considering the different functions of different macrophage phenotypes, hepatic IRI can be improved by regulating macrophage polarization. In this study, we found that liraglutide reduced hepatic IRI and inhibited the inflammatory response during IRI. This protective effect was achieved *via* the regulation of macrophage polarization by GLP-1R.

The liver, the largest immune organ in the human body, contains metabolically active hepatocytes, non-hepatocyte parenchymal cells, and various immune cell populations. These cells play an important role in hepatic IRI (25, 26). To confirm the cells on which liraglutide acts, we first investigated the effect of liraglutide on hepatocytes *in vitro*. However, liraglutide did not show any protective effect against hypoxia-induced hepatocyte apoptosis (data not shown), which may be attributed to the lack of GLP-1R expression in hepatocytes (27, 28). Next, we removed KCs, NKs, NKTs, and neutrophils from a mouse hepatic IRI model. Interestingly, the protective effect of liraglutide on hepatic IRI disappeared after macrophage depletion; however, after the removal of NKs, NKTs, and neutrophils, liraglutide still exerted a good liver protection effect. These results indicate that the protective effect of liraglutide against hepatic IRI may be achieved through their action on macrophages.

The liver contains the largest proportion of macrophages among parenchymal organs, and macrophages play a crucial role in maintaining liver homeostasis and pathology (29, 30). Macrophages can change their phenotype according to changes in the surrounding microenvironment, and phenotypic variation in macrophages affects their ability to secrete a variety of cytokines (31). Both phenotypes can modulate sterile liver inflammation and play important roles in initiating, maintaining, and ameliorating hepatic IRI. Macrophages can be induced to differentiate into the M1 type upon lipopolysaccharide stimulation. M1 macrophages highly express CD80, CD86, MHC II, and iNOS and secrete pro-inflammatory factors (30–32). In this study, the serum levels of ALT, AST, and pro-inflammatory cytokines, including TNF-α, IL-1β, and IL-6, significantly decreased after liraglutide treatment. Next, we examined macrophage polarization markers in liver tissues *via* flow cytometry. As expected, liraglutide treatment reduced the I/R-induced activation and recruitment of M1 macrophages. M2 macrophages play a crucial role in hepatic IRI as an anti-inflammatory phenotype. Therefore, we investigated the effects of liraglutide on M2 macrophages. Unfortunately, liraglutide treatment did not promote the growth of M2 macrophages (data not shown).

Most of the effects of liraglutide are mediated by GLP-1R, which is expressed in different cells and tissues. In the heart, GLP-1R is expressed on cardiac graft endothelial cells, and by activating the GLP-1R receptor on endothelial cells, it can delay the occurrence of graft CVD in a mouse heart transplant model (15). GLP-1R activation in tubular epithelial cells in the kidney reduces HMGB1 release during renal IRI, thereby attenuating renal injury (33). Recent studies have shown that GLP-1R is expressed in the liver (12). In a model of nonalcoholic fatty liver disease (NAFLD), liraglutide could regulate the transformation of macrophages, thereby reducing lipid accumulation and steatosis (34). However, little research has been conducted on the relationship between liraglutide pretreatment and macrophage polarity. Therefore, we speculate that liraglutide may attenuate hepatic IRI by modulating the phenotype of macrophages *via* acting on GLP-1R. Our study found that in GLP-1R^-/-^ mice, the protective effect of liraglutide against hepatic IRI was partly diminished. In the *in vitro* study, we assessed the role of liraglutide in macrophage polarization by examining the expression profiles of M1 in BMDMs from GLP-1R^-/-^ and C57BL/6J mice. Consistent with the *in vivo* study, liraglutide attenuated LPS-induced M1 polarization and decreased the expression of M1 markers. However, the inhibitory effect of liraglutide on LPS-induced M1 polarization was largely abolished in BMDMs from GLP-1R^-/-^ mice, and the regulatory effect of liraglutide on macrophages may be mediated by the STAT1 signaling pathway. Our data derived from GLP-1R-/- and C57BL/6J mice clearly indicated that the regulation of macrophage polarization by liraglutide is dependent on the GLP-1R receptor.

However, our study has some limitations. First, as the largest immune organ in the human body, the liver contains many immune cells; however, we only investigated the effects of liraglutide on neutrophils, NKs, NKTs, and macrophages during hepatic IRI, and whether liraglutide affects other cells in the liver remains to be determined. Second, the protective effects of liraglutide on liver IRI were partially reversed in GLP-1R^-/-^ mice, indicating that liraglutide may act through non-receptor pathways. Third, a recent study indicated that metabolic reprogramming is involved in macrophage polarization, and metabolic and intrinsic hormonal changes were observed in ischemic patients (35, 36); whether liraglutide, as an intrinsic hormone analog, affects macrophages by regulating metabolic reprogramming needs further investigation. These limitations should be addressed in future studies.

In summary, our study shows that liraglutide inhibits macrophage polarization towards an inflammatory phenotype *via* the GLP-1R receptor. Furthermore, our findings indicate that liraglutide pretreatment attenuates hepatic IRI and improves liver function. These results may provide a new direction for the clinical treatment of HIRI.

## Data Availability Statement

The original contributions presented in the study are included in the article/supplementary material. Further inquiries can be directed to the corresponding authors.

## Ethics Statement

The animal study was reviewed and approved by Tongji Medical College Committees on Animal Experimentation.

## Author Contributions

Author contributions: JY had full access to all the data in the study and was responsible for the integrity of the data and accuracy of the data analysis. Study design: CD, S-LL, and Z-MW. Acquisition of data: CD, CX, and F-HC. Analysis and interpretation of data: CD, X-FH, and RC. Drafting of the manuscript: S-LL, Z-MW, CD, H-BS, Y-NX, and YQ. Critical revision of the manuscript for important intellectual content: CD and JY. Statistical analysis: S-LL, Z-MW, and JY. Obtaining funding: JY, CD, and Z-MW. Administrative, technical, or material support: H-BS and BL. Supervision: JY. All authors contributed to the article and approved the submitted version.

## Funding

This study was supported by the National Natural Science Foundation of China (grant numbers 81873624, 81900368, and 82100800).

## Conflict of Interest

The authors declare that the research was conducted in the absence of any commercial or financial relationships that could be construed as a potential conflict of interest.

## Publisher’s Note

All claims expressed in this article are solely those of the authors and do not necessarily represent those of their affiliated organizations, or those of the publisher, the editors and the reviewers. Any product that may be evaluated in this article, or claim that may be made by its manufacturer, is not guaranteed or endorsed by the publisher.
